# Method for optimizing imaging parameters to record neuronal and cellular activity at depth with bioluminescence

**DOI:** 10.1117/1.NPh.11.2.024206

**Published:** 2024-03-28

**Authors:** Alexander D. Silvagnoli, Kaylee A. Taylor, Ashley N. Slaviero, Eric D. Petersen

**Affiliations:** a Central Michigan University, College of Medicine, Mount Pleasant, Michigan, United States; b Central Michigan University, Program in Neuroscience, Mount Pleasant, Michigan, United States; c Central Michigan University, Biochemistry, Cell and Molecular Biology Graduate Program, Mount Pleasant, Michigan, United States

**Keywords:** bioluminescence, activity imaging, phantom, optogenetics

## Abstract

**Significance:**

Optical imaging has accelerated neuroscience in recent years. Genetically encoded fluorescent activity sensors of calcium, neurotransmitters, and voltage are commonly used for optical recording of neuronal activity. However, fluorescence imaging is limited to superficial regions for *in vivo* activity imaging, due to photon scattering and absorbance. Bioluminescence imaging offers a promising alternative for achieving activity imaging in deeper brain regions without hardware implanted within the brain. Bioluminescent reporters can be genetically encoded and produce photons without external excitation. The use of enzymatic photon production also enables prolonged imaging sessions without the risk of photobleaching or phototoxicity, making bioluminescence suitable for non-invasive imaging of deep neuronal populations.

**Aim:**

To facilitate the adoption of bioluminescent activity imaging, we sought to develop a low cost, simple *in vitro* method that simulates *in vivo* conditions to optimize imaging parameters for determining optimal exposure times and optical hardware configurations to determine what frame rates can be captured with an individual lab’s imaging hardware with sufficient signal-to-noise ratios without the use of animals prior to starting an *in vivo* experiment.

**Approach:**

We developed an assay for modeling *in vivo* optical conditions with a brain tissue phantom paired with engineered cells that produce bioluminescence. We then used this assay to limit-test the detection depth versus maximum frame rate for bioluminescence imaging at experimentally relevant tissue depths using off-the-shelf imaging hardware.

**Results:**

We developed an assay for modeling *in vivo* optical conditions with a brain tissue phantom paired with engineered cells that produce bioluminescence. With this method, we demonstrate an effective means for increasing the utility of bioluminescent tools and lowering the barrier to adoption of bioluminescence activity imaging.

**Conclusions:**

We demonstrated an improved method for optimizing imaging parameters for activity imaging *in vivo* with bioluminescent sensors.

## 1 Introduction

Both single and multiphoton fluorescence imaging suffer difficulties when acquiring signal from deep within the brain. Single photon imaging is typically restricted to superficial layers of cortex, with a recommended max signal resolution depth of <200  μm.^1^^,^^2^ Beyond this depth, the true signal becomes difficult to resolve from background values due to scattering, autofluorescence, and photon energy loss. Multiphoton microscopes enable imaging at greater depths while achieving single cell resolution. Two and three photon imaging methods can achieve depths of 1200  μm and 1700  μm, respectively, while providing single cell resolution.^3^^–^^5^ Two-photon microscopy is well suited for imaging the cortex but experiences significant scattering effects as a function of depth.^6^^,^^7^ Although three-photon microscopy can image deeper than the cortex, it is worth noting that the associated equipment costs may be challenging for many labs to accommodate. For these reasons, the visualization of sub-cortical neuronal population dynamics continues to prove challenging or inaccessible for many labs.

Optical recording in deeper brain regions has been achieved with fluorescence using techniques, such as fiber photometry without single cell resolution. By exciting the genetically fluorescent sensor through a light fiber, activity can be recorded from a group of neurons near the tip of the fiber as a bulk population signal. This increases the accessibility to deeper regions; however, it comes at the cost of a significant disturbance to brain tissue as well as limited population sampling.^8^^,^^9^ The population size available for fiber photometry is limited to cells near the tip of the fiber. Pisanello et al. demonstrated that the effective volume of recording for a standard Ø400  μm, 0.50 NA optical fiber was a $10^{6}\;{\mu m}^{3}$ extending 200  μm in front of the fiber.^10^ A fiber of similar dimensions to the provided example would be expected to displace a volume of ∼0.25 to $0.5\;{\mathrm{mm}}^{3}$ when inserted between 2 to $4\;{\mathrm{mm}}^{3}$ deep, respectively.^11^ Also worth consideration are the effects of an implant within the tissue of a freely moving animal. The fiber employed for this type of imaging is typically stiffer than the surrounding tissue with few notable exceptions.^12^^,^^13^ This resistance of the fiber to movement of the surrounding tissue can introduce disruptive forces to local vasculature, affecting both the blood brain barrier and parenchyma.^12^^,^^14^ In freely behaving animals, this can introduce damage that cannot be accounted for until the end of the experimental period, especially for animals used for repeated recordings over time.

Gradient refractive index lenses, or GRIN lenses, have gained popularity for activity imaging for their advantages over fiberoptic implants in providing single cell resolution. These lenses focus light by changing the refraction of light within the lens, providing a focused beam of photons on the subject.^15^^,^^16^ However, they are not without limitations. The implantation of a lens is no less invasive than optical fiber. Recording from a larger population of neurons can require a lens of 0.5 –to 2 mm in diameter, displacing a tissue volume of ∼1.6 to $25\;{\mathrm{mm}}^{3}$ with the lens alone.^17^ However, the total amount of tissue displaced is 2.8× greater than the volume of the lens. This is because the wall thickness for the glass tube accompanying the lens displaces yet more tissue. For example, the guide cannula for a 0.5 mm GRIN lens can be 0.84 mm in diameter.^18^ As is a common drawback to most brain implants, implanted fibers and lenses can induce glial scarring, a condition in which reactive astrocytes can encapsulate the implant, decreasing the signal quality.^19^^,^^20^

Bioluminescence imaging offers an opportunity for deep brain activity data collection without the use of implants.^15^^,^^21^ Both terrestrial and marine versions of luciferase enzymes are used in a wide range of life science fields including neurobiology and oncology.^16^^,^^22^^,^^23^ Bioluminescent light is produced by enzymatic oxidation of a substrate, referred to as a luciferin.^24^ By generating light within the tissue, it is possible to collect signal from deep structures without an implant, at the cost of lower spatial and temporal resolution compared with fluorescence imaging. Additionally, the production of light via consumption of substrates eliminates the possibility of photobleaching and has not been shown to have potential for phototoxicity. Thus, long imaging periods over repeated sessions can be leveraged to track long-term activity, a long-standing goal of neuroscience. Bioluminescent indicators can also be selectively expressed utilizing selective delivery methods such as cell type specific or neuron subtype specific promoters and selective viral capsids. The specificity of expression coupled with in situ light generation simplifies the imaging requirements for capturing high signal to noise data, obviating the need for sophisticated optics due to the low hardware requirements for bioluminescence imaging.^25^ Significant developments in the field of bioluminescence imaging have enabled signal detection from deep structures such as the hippocampus,^15^^,^^21^ basal ganglia,^16^^,^^22^^,^^23^ hypothalamus,^26^ and spinal cord.^27^^,^^28^ Bioluminescence has also been imaged from implanted cells in freely behaving marmosets without surgical implants or attached hardware at a depth of 4.8 mm^29^ and enabled bioluminescence based voltage and calcium imaging in freely behaving mice without implants or any attached hardware.^30^^,^^31^

Recently, bioluminescent sensors that can report calcium, voltage, and neurotransmitter release, events crucial to neuronal communication and information processing, have been developed. Genetically encoded bioluminescent indicators are favorable for deep tissue imaging due to the lack of endogenous light emission in mammalian tissue. This means that a low photon output should provide high SNR for transient calcium events. For example, Oh et al. utilized a red shifted bioluminescent calcium indicator (CamBI) to image the activity of the whole liver, imaging in the intact animal.^32^ Tian et al. was able to record calcium activity in the basolateral amygdala through the closed skull utilizing their red shifted bioluminescent calcium sensor BRIC with a modified luciferase pro substrate, achieving bioluminescent activity imaging at a depth of 5 mm, which is far below the effective fluorescence imaging range without an implanted lens or fiber.^33^ Lambert et al. recently developed a green bioluminescent calcium sensor based on the new luciferase Sensor Scaffold Luciferase (SSLuc), CaBLAM, with an unparalleled dynamic range that we expect to enable more robust responses to be optically recorded in deep brain regions.^34^ We have also recently expanded genetically encoded bioluminescent sensors to include the detection of neurotransmitters *in vivo*, developing bioluminescent indicator of glutamate (BLING), which emits blue light and demonstrated its ability to detect seizure activity in the rat at a depth of 2 mm while imaging through the closed skull.^35^

The promise shown in previous applications of bioluminescence activity imaging is promising, but a barrier to widespread adoption is changing imaging approaches to accommodate the low photon output when imaging through tissue. The scattering and absorbing properties of brain tissue limit the ability of photons to travel from their point of origin unchanged.^36^ Computational and empirical calculations of the mean free path (MFP) determine that, for a photon of a given wavelength, the distance that the photon can travel without obstruction is approximately 90  μm.^37^^,^^38^ Interference accumulated as a function of distance results in scattering, limiting the ability to resolve individual sources. Deep imaging of tissue is therefore restricted to the characterization of whole neural populations rather than individual cells due to scattering and absorption. To compensate for limited photon production and decreased photon escape with increasing target depth, exposure times are lengthened, sacrificing temporal resolution for increased SNR to achieve non-invasive deep brain activity imaging.

Here, we present a method for optimizing imaging parameters with bioluminescence using a tissue phantom adapted from Ntombela et al. that simulates the absorbing and scattering properties of brain tissue to enable adoption of bioluminescent sensors for activity imaging. We paired this with a biological light source and demonstrated the ability to optimize imaging parameters without necessitating the use of animals to determine the detection limits of imaging hardware. This approach can be used to determine what timescale can be recorded using various imaging setups that a lab may have to facilitate *in vivo* experimental designs. Our goal is that researchers will be able to use this approach to determine what types of neuronal events and timescales can be captured prior to initiating animal experiments using an inexpensive optical brain phantom. We translate *in vitro* imaging conditions to *in vivo*-like conditions, enabling the approximation and optimization of *in vivo* imaging without significant animal use. To validate this approach, we employ the same imaging parameters *in vivo*. From these results, we expect that it is possible for others to easily adopt this approach for imaging hardware optimization and to determine if physiological timescales can be recorded that align with experimental goals prior to initiating *in vivo* experiments, saving time and resources, and reducing animal use.

## 2 Materials and Methods

### 2.1 Optical Tissue Phantom

We chose an optical phantom recipe from a previous publication by Ntombela et al. that was designed with the intent of replicating the light absorbing properties of multiple kinds of soft tissue. Their phantom was tested with multiple component ratios and validated with laser absorbance.^39^ We used the ratio of components best suited for replicating brain grey matter optical properties. 2 grams agar (BD Ref. 214530) and 300 mg of aluminum oxide $\left[{\mathrm{Al}}_{2}O_{3}\right]$ (Sigma Aldrich, 199443) were weighed and mixed in an Erlenmeyer flask containing 100 mL de-ionized water and a magnetic stir bar. The mixture was heated while stirring until the temperature reached 94°C. Temperature was monitored continuously using ThermPro Food Thermometer (Model No. TP-02). Once reached, the mixture was moved to a separate stir plate at 55°C to prevent early firming. 300  μL India ink (Bombay, BOMB10OZS7BY) was added and stirred until completely mixed.

The mixture was then pipetted into 96 well plates for molding. Plates containing molten phantom were covered with parafilm and the original lid to prevent loss of moisture while firming. Each plate was placed at 4°C to rapidly solidify to prevent precipitation of the aluminum oxide and then was left for at least 24 h. Agar-based brain phantoms shaped like pucks were later removed from the plates via hydraulic pressure forced from the well’s bottom. To accomplish this, a 30-gauge hypodermic needle was attached to a 3 mL syringe containing 1× phosphate buffered saline (PBS). The needle was inserted at the periphery of a phantom puck, carefully to not puncture the puck, and PBS was injected into the bottom of the well until the puck was fully ejected. Using this technique, we were able to leverage the generated pressure to force the puck out of the well without damaging it.

The solidified phantom pucks with lengths of 2, 4, and 6 mm were cut using a spinal cord steel matrix (Alto SA-5110) and stainless-steel single edge razor blades (VWR 55411-050). This instrument was utilized simply due to already having it, and we expect many other means can be used to accomplish this task with sufficient precision. To control potential variability, pucks for a given “depth” were weighed, and a running average was established. This average was later used to preemptively remove potential outliers. Pucks differing by more than 2 mg from the average for a given “depth” were disposed of prior to the imaging experiment.

### 2.2 Evaluating the Optical Tissue Phantom Against Brain Tissue

Pucks of 2, 4, and 6 mm depths were cut and placed into black clear bottom 96-well plates (Corning Ref. 3603). Experiments were performed using replicates of five per given depth. Ink concentration was varied according to a 1:2 serial dilution for comparison of ink concentration versus depth.

Brain tissue was harvested from mice C57BL/6 wild type mice (age > P60) with sufficient tissue to run absorbance replicates in triplicate. Animals were euthanized using ${\mathrm{CO}}_{2}$ followed by cervical dislocation, and the brain was immediately removed and put in PBS chilled on ice. Mice were deliberately not perfused. Brain tissue samples were divided into three groups: unfixed, 24-h fixation, and 48-h fixation. Fixed brains were placed in 4% paraformaldehyde in PBS at 4°C. Unfixed brains were extracted and used the same day. Brain tissue samples were sectioned into lengths of 2, 4, and 6 mm. Brain samples were placed into wells in replicates of three alongside the phantom pucks. Wells containing PBS were used as a blank control.

Absorbance spectra were generated using a multipoint 3×3 well scan of each well from 450 to 700 nm with a plate reader (Tecan Spark). Preliminary experiments determined that there was a significant difference in absorption by the phantom pucks when the absorbance was read at room temperature (approx. 27°C) and 37°C.

### 2.3 Bioluminescence Imaging Through the Phantom

#### 2.3.1 Acute transfection with SSLuc

Hek 293 cells were in a 24-well plate, grown to 70% confluency. Each well was then treated with Lipofectamine 2000 (Thermofisher Cat No. 11668-019) [2  μL/well] and SSLuc plasmid [0.8  μg/well]. Media was changed after 4 h and cells incubated for 48 h before testing.

#### 2.3.2 IVIS imaging: bioluminescent cells

Acutely transfected HEK293 cells expressing SSLuc were suspended and concentrated in FluroBrite DMEM to $6\times 10^{6}\;\text{cells}/\mathrm{mL}$ (Ref. A18967-01). 35  μL of cells (∼200,000) were added to wells spaced two rows and two columns apart. 5  μL of 2 mM h-Coelenterazine (hCTZ) was added to wells containing cells just prior to being placed in an IVIS Lumina. Pucks produced as described previously were placed over wells containing cells in the IVIS machine and imaged at 100, 250, 500, 1000, and 2000 ms exposure times. The binning was set to medium, and the F-stop was set at 1. The height of the camera was set to 1.5 cm. The temperature for the IVIS was set at 37°C.

#### 2.3.3 Stable cell line generation

Flp-In-293 cells (ThermoFisher Scientific Ref. R75007) were transfected according to standard lipofection procedure (Lipofectamine 2000 ThermoFisher Scientific) with a plasmid containing the intact bioluminescent luciferase SSLuc that was used to create the calcium sensor CaBLAM and a Hygromycin B resistance gene.^34^ Cell media was changed 6 hours after transfection, and cells were split the next morning and seeded at ∼25% confluency on a new plate. Three days post transfection, cells were treated with Hygromycin B (Invitrogen Ref. 10687010) [100  μg/mL] with DMEM, 10% FBS, 1x Pen/Strep. Media was changed, and fresh Hygromycin B was added every three days until cell colonies had formed. Hygromycin resistant colonies were pooled and expanded. Single cells were then selected by performing a dilution series on the polyclonal pool and seeding approximately one cell per well on a 96-well plate. Cultures derived from monoclonal colonies were evaluated in replicates of 5 in 96 well plates for consistency of expression and brightness in response to h-Coelenterazine (hCTZ) [250  μM] (NanoLight, Ref. #301) administration, read with a Tecan Spark plate reader.

#### 2.3.4 Bioluminescence microscope setup

We assembled off-the-shelf components likely to be standard equipment for many labs. We mounted an Andor EMCCD camera, xIon 888 onto a dissection microscope (Leica No. MZ10 F) with a 1.0× objective and a tube lens adaptor (Leica No. 10447367). Our setup was surrounded by a homemade dark box constructed of 1/2-inch PVC tubing found at most hardware stores, covered in a black drop cloth (Thor Labs Ref. BK5). To ensure that the dark box was not permissive of light, we tested the inside of the box using a light power meter (Thor Labs Ref. S121C). Recorded values were found to not be significantly different from background noise of the light power meter when covered with the room lights off. This was interpreted to mean that the dark box surrounding the microscope was light tight.

#### 2.3.5 Bioluminescence microscope imaging: bioluminescent cells

Confluent Flp-In-SSLuc cells were suspended and concentrated as described above. hCTZ was also prepared as described above. Phantom pucks of 2, 4, and 6 mm were cut and placed in PBS to prevent dehydration. Prior to recording, 100 frames of dark background were recorded for later analysis. Wells were centered in the field of view, and an image was taken to use as a reference for ROI placement. Each well was prepared and recorded individually. Recorded wells were spaced two wells apart vertically and horizontally. Stocks of cells and hCTZ were generated and kept at 37°C in a warm water bath for the duration of testing. 30  μL of cells and 30  μL of hCTZ [0.5 mM] were mixed in microcentrifuge tubes and pipetted up and down multiple times to ensure even mixing. 40  μL of this mixture was placed into a well and covered immediately with a phantom puck. The drape covering the light proof box over the microscope was secured over the dark box. Each sample was recorded for 200 frames at 95, 100, 150, 250, 500, 1000, and 2000 ms exposures.

#### 2.3.6 Bioluminescent titration assay

HEK 293 cells stably expressing SSLuc were cultured in large quantities for titration experiments ($n\geq {8e}^{6}\;\text{cells}$). Cells were then resuspended and concentrated into 1 mL volumes of artificial cerebral spinal fluid [ACSF, micro-osmolarity 310±5, pH 7.4] (made as Berglund 2016).^40^ Dilutions were generated using a 1:2 dilution strategy for cell concentrations of 200 k, 100 k, 50 k, 25 k, and 12.5 k cells per well. Cell concentrations were verified via hemocytometer. Simultaneously, a 667  μM stock of hCTZ was made by adding 7.5  μL of 50 mM hCTZ to 1.5 mL ACSF. hCTZ was mixed with pipetting until evenly distributed.

To image each sample, 35  μL of cells was added to an open PCR tube (Thermo Scientific Mfr No. AB0773). 15  μL of stock hCTZ was added and mixed gently with a pipette 5×. 40  μL of this mix was added to a given well. The well was then covered with a 2 mm brain phantom puck. The dark cover was placed over the microscope and EMCCD and secured. Samples were imaged for 100 frames per given exposure.

#### 2.3.7 Data processing: titration assay

Due to the large volume of images produced by the assay, it was not feasible to process all of the images by hand. Instead, we wrote a custom macro using FIJI to batch process images. First, we generated “dark images” manually. This was done by processing recordings of empty, dark backgrounds with the same parameters as experimental groups. Each recording was 100 frames. Dark recordings were made for each exposure time. Recorded dark frames were processed for outlier removal (x and y pixel radius = 40, std. dev = 3.0). A gaussian blur (sigma = 2.0) was applied to all frames. Following this step, each stack of images was projected via their median and saved.

A similar process was used to develop the images of experimental wells with some key additions. After the Gaussian blur step, experimental image stacks were projected as a sum of their slices. A threshold was then applied automatically using the triangle method. We found that this threshold captured the signal best, especially for lowlight images. The threshold was used to generate a mask that was saved as an 8-bit image. We then applied the particle counting function to capture the mask and add it to the ROI Manager as a custom ROI. The custom ROI was added back into the stack that had been processed up to the Gaussian projection step. Stacks were measured with custom ROIs generated for that stack. Metrics analyzed included the area, mean gray value, standard deviation, integrated density, and raw integrated density. Raw integrated density was extracted from each measurement output and collated into a single .csv file. Statistical analysis was conducted with Prism software.

### 2.4 *In Vivo* Data Collection

#### 2.4.1 Viral transduction

All experiments involving animals were conducted in accordance with Central Michigan University IACUC and lab safety guidelines. C57BL/6 mice between 4 and 8 months were injected unilaterally using AAV-*hSyn*-ssLuc at stereotaxic coordinates [A/P=(−1.0),M/L=(−2.5)] relative to bregma. Prior to surgery, mice were anesthetized using isoflurane at 2.0%. A small burr hole was created above the coordinates using a dental drill. Using a World Precision Instruments syringe with a 33-gauge needle (NanoFil Ref. NF33BV), mice were injected with 1  μL of virus [${3\times 10}^{13}\;\mathrm{vg}/\mathrm{mL}$] at 2 or 4 mm of depth relative to the top of the cortex at a rate of 0.05  μL/min. Following completion of the injection, the needle was allowed to sit at depth for 10 min. A small dab of dental cement was used to seal the burr hole and the skin was closed with sutures. Mice were treated with triple-antibiotic ointment and lidocaine.

#### 2.4.2 Skull thinning

After 2 weeks, the skull covering the injected hemisphere was thinned. Mice were anesthetized with isoflurane. A dental drill was used to gently shave away bone tissue from the injected side until only cortical bone remained,^41^ and the wound was sutured closed. Mice were allowed to recover from surgery in a heated chamber at 37°C until grooming was observed. A 48-h recovery period followed the procedure.

#### 2.4.3 Animal imaging with IVIS

Thirty minutes prior to anesthesia, animals were individually injected IP with water soluble hCTZ (NanoLight Cat#3011). Each was anesthetized using a ketamine (10  mg/kg) and xylazine (2  mg/kg) cocktail and tested for toe and tail pinch response. The skin above the thinned skull was re-opened and pulled to either side using surgical clips. Emitted bioluminescence was recorded through the exposed, thinned skull using the same parameters as the plates with phantoms at 100, 250, 500, 1000, and 2000 ms. Animals were then allowed to recover in a heated recovery chamber until alert.

#### 2.4.4 Animal imaging with EMCCD

Similar to the IVIS imaging, animals were injected with hCTZ (10  mg/kg) and then anesthetized with a ketamine/xylazine cocktail. After checking for toe and tail pinch response, the thinned skull above the injection site was exposed and surgical clips were used to pull the skin aside. Mice were placed on a heated pad, and bioluminescence was recorded at the exposure times. Mice were placed on a heated pad, and bioluminescence was recorded at 100, 150, 250, 500, 1000, and 2000 ms. A long exposure was taken at the end of the recording period to validate the presence of bioluminescence. Mice were perfused immediately at the end of their recording session.

### 2.5 Bioluminescent Glutamate Sensor Assay

HEK 293 cells were seeded in two T25 flasks at $1.2\times 10^{6}$ each for transfection with BLING 1.0. Eight hours post transfection, cells were lipofected following manufacturer instructions. 20  μL lipofectamine 2000 was mixed with 0.5 mL Opti MEM and 8  μg plasmid DNA. This was then added to cultured HEK293 cells. The media was changed after 4 h of incubation, and cells were incubated for 48 h before imaging. Cells were mechanically dissociated to prevent an enzymatic breakdown of the surface displayed sensor protein and resuspended in 1 mL of Tris buffered saline (TBS). An 8 mM glutamate solution was prepared using monosodium glutamate in TBS, and a 2.5 mM hCTZ solution was prepared by diluting a 50 mM stock in TBS. Pucks measuring 2 mm in depth were made and cut as described above. For each recording, 30  μL of cells (100,000) were mixed with 5  μL of hCTZ and 5  μL of buffer or with 5  μL of hCTZ and 5  μL of glutamate before being added to a single well of a black, opaque-bottom 96 well plate and covered with a phantom. Recordings were taken in replicates of five with 100 frames each at 95, 150, 250, 500, 1000, and 2000 ms exposures. Following the collection of bioluminescence from each replicate, images were processed using the same method for processing our titration assay. Data were consolidated from each CSV file generated by the script and pasted into the Prism software for analysis. Each ROI generated for the BLING-Phantom replicates was visually inspected to ensure accuracy. There were no major aberrations in the ROIs (i.e., non-circular masks or placement of ROI far outside of reference location).

## 3 Results

### 3.1 Absorption Spectra of Phantom Versus Brain Tissue

**Fig. 1:**
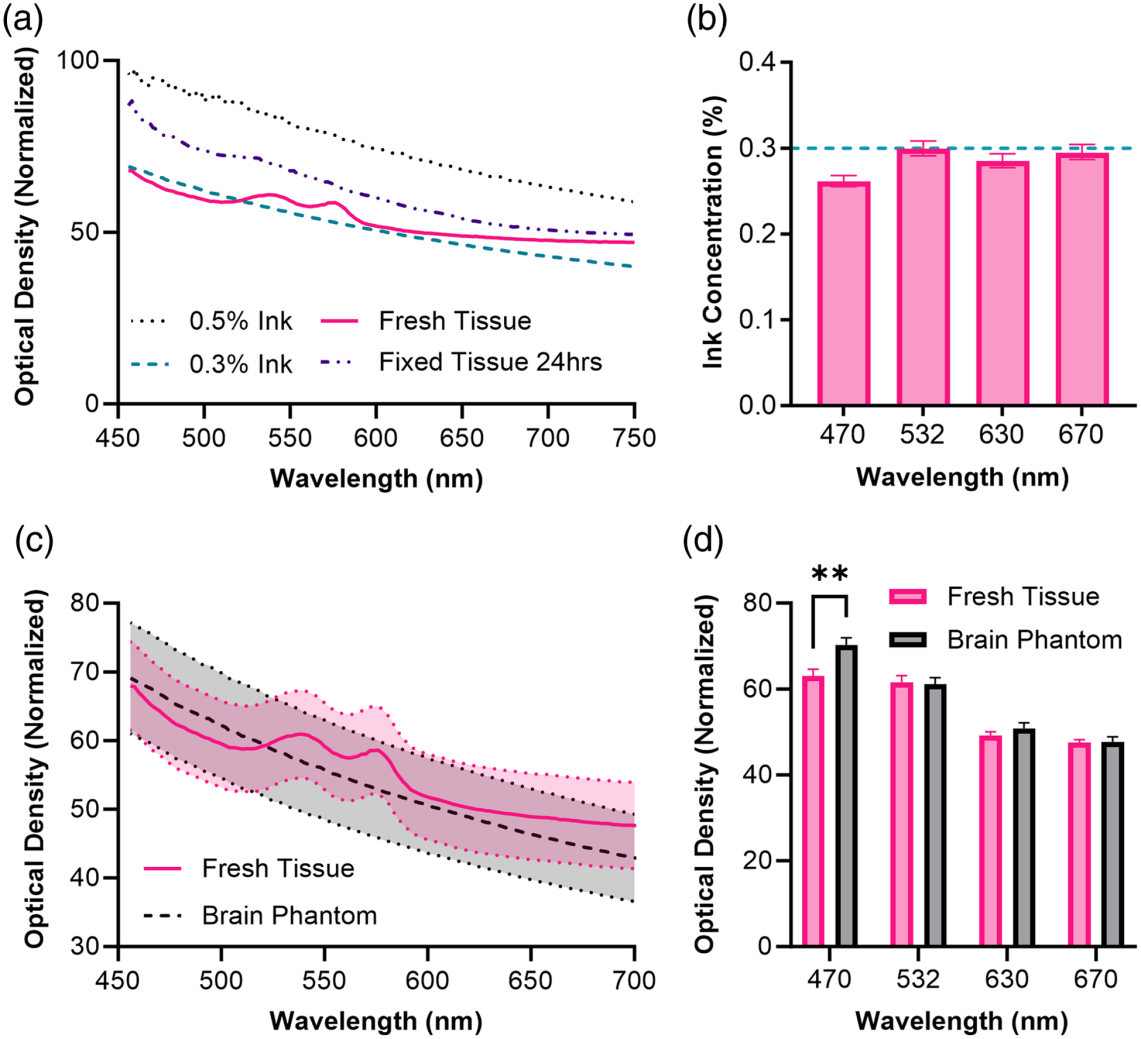
Evaluation of the phantom as a proxy for brain tissue. (a) Plot of the absorbance of phantom pucks against wavelength. (b) Interpolated ink concentrations of brain tissue based on the absorbance curves and regression modeling for the wavelength of interest. The dotted line indicates the ink concentration 0.3% reproduced from Ntombela et al. to match the absorbance of grey matter. (c) Absorbance plotted for the brain phantom versus fresh tissue illustrates the overlap between fresh brain tissue and the brain phantom at 0.3% India ink concentration. (d) Comparing the absorbance values of fresh brain tissue versus the phantom at 470, 532, 630, and 670 nm. No significant differences shown for each wavelength as determined with two-way ANOVA except for 470 nm (p<0.01).

By measuring the absorbance of the brain phantoms, we determined that the ink concentration reported to correspond to brain tissue in Ntombela et al. closely matches the absorbance curve that we generated, compared with freshly harvested mouse brain tissue but not fixed brain tissue [Fig. 1(a)].^39^ Interestingly, because our coverage of the visible light spectrum included several points between those tested in Ntombela et al., we were able to observe a bump in the fresh tissue absorbance from 500 to 600 nm [Fig. 1(c)]. These results agree with the absorbance of hemoglobin in fresh tissue.^42^ This was done to simulate *in vivo* optical conditions as closely as possible. The examination of the curves for fresh tissue and the 0.3% India ink phantom shows a close overlap throughout most of the visible light spectrum. An analysis using two-way ANOVA showed no statistically significant difference between the phantom and fresh tissue at the wavelengths of interest except for 470 nm (p<0.01) [Fig. 1(b)].

To further validate that our findings were consistent with expectations, we back-calculated hypothetical ink concentrations based on the observed optical density (OD) of fresh tissue. Using non-linear regression modeling with Prism 9, the decay of optical density in the phantom as a function of increased wavelength was generated for the phantom containing 0.3% ink ($R^{2}>0.95$) [Fig. 1(d)]. OD values of brain tissue at critical wavelengths were then interpolated along the curve to determine the approximate “ink concentration” required to match that of fresh brain tissue, of which 470 nm would require significantly lower ink, expected to be 0.265 [Fig. 1(b)]. Here we found that it was possible to closely model the absorbance of light by brain tissue using the phantom alone. We believe that this is significant due to the extreme simplicity of the phantom itself relative to the natural complexity of optimizing imaging experiments with animal models. This was encouraging as our aim was to reduce the overall time and financial cost of optically modeling the brain.

### 3.2 Evaluation of IVIS Imaging System for Depth Comparison

**Fig. 2:**
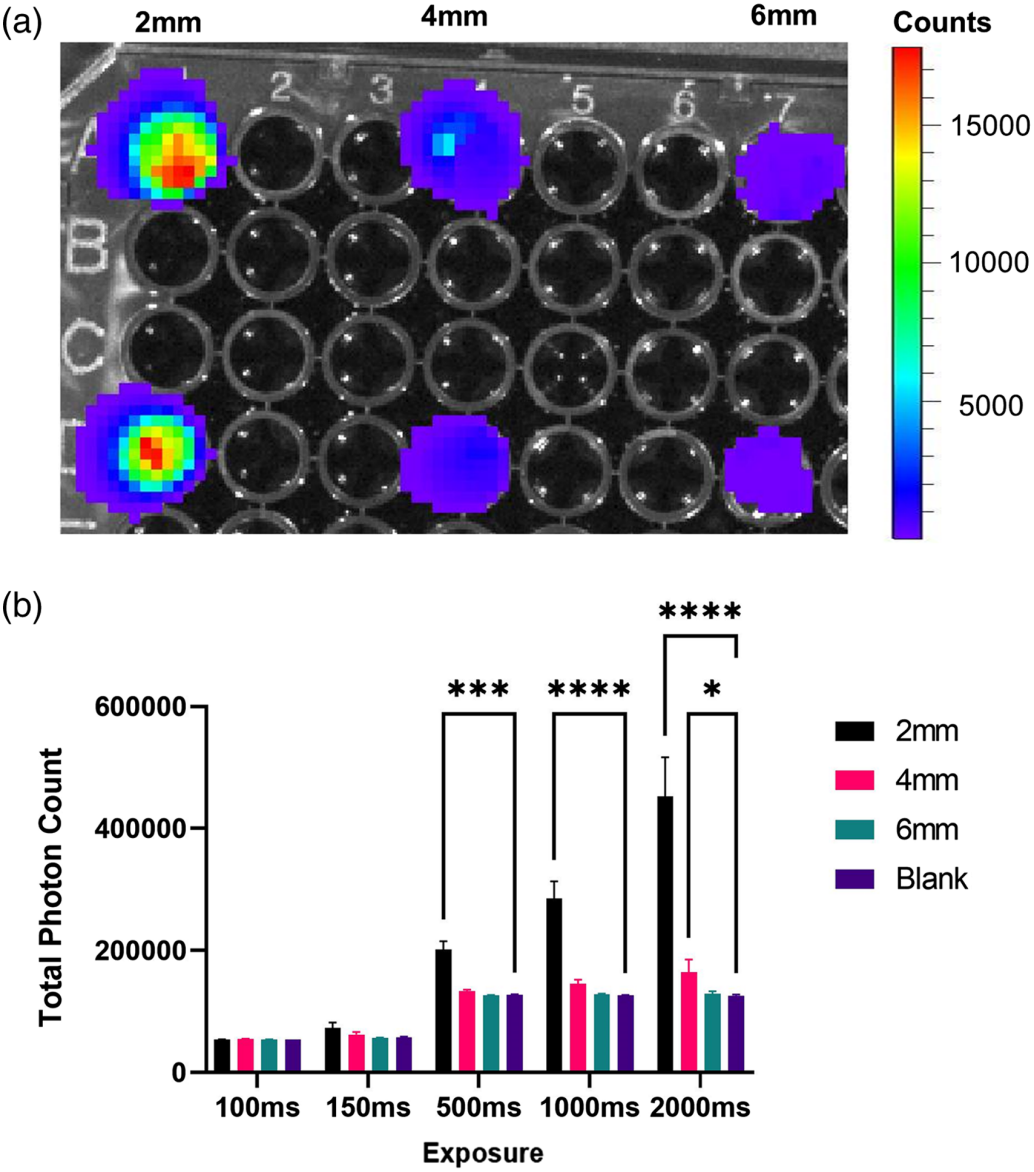
Testing the brain phantoms with an IVIS. (a) A representative image of bioluminescence from our engineered cells recorded through phantoms of varying thicknesses. From left to right, the wells are occupied by 2, 4, and 6 mm phantom pucks. (b) Measured values of photon count through the tissue phantom. Photons were generated from HEK 293 cells expressing a green-shifted bioluminescent constructed emitting photons at the 535 nm wavelength.

We combined our phantom with an opaque bottom black 96 well plate (Corning Ref. 3650) to simulate light generation from within the brain as closely as possible. Phantom pucks were placed on top of small volumes of media containing bioluminescent cells treated with hCTZ. Wells were tested in duplicates of 2, 4, and 6 mm puck depths with additional wells for blank (i.e. non-treated) controls [Fig. 2(a)]. Our data showed that it was possible to collect bioluminescent light through the tissue phantom at significant levels above background. However, a two-way ANOVA analysis of wells containing transfected HEK 293 SSLuc cells treated with hCTZ did not show statistical significance against background until after 500 ms of exposure [Fig. 2(b)]. In our case, we were unable to distinguish between signal and background at or below 250 ms with the conditions used in our experiment [Fig. 2(b)]. This could be partly due to the included Living Image software and how images are processed and corrections are applied.

### 3.3 Titration of SSLuc Expressing Cells

**Fig. 3:**
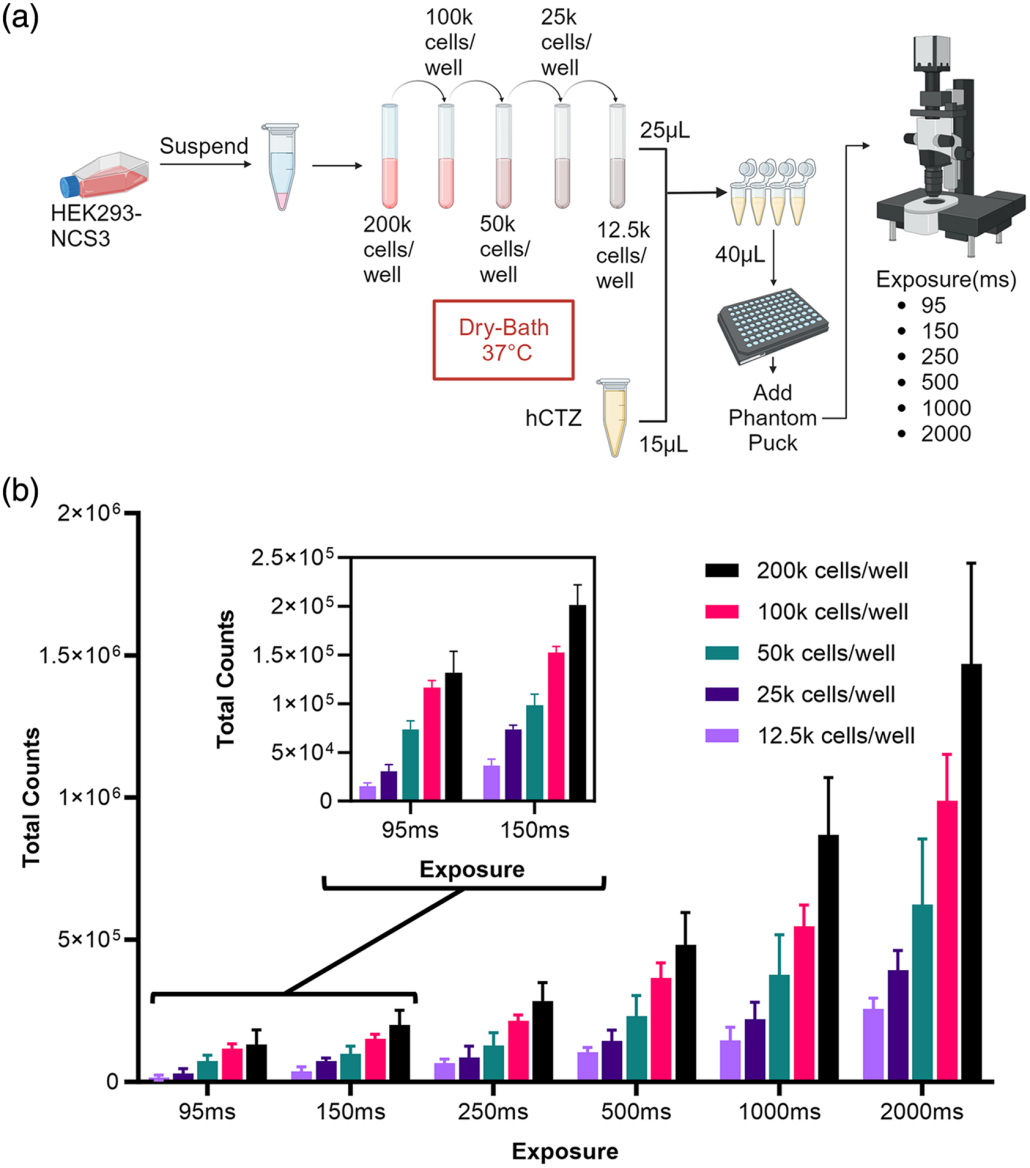
Titration of bioluminescence emitting cells. (a) A diagram of the workflow illustrating the method of bringing the cells from culture to testing. HEK 293 SSLuc cells were diluted from stock concentration in 1:2 serial dilution. Each dilution was tested for bioluminescence in replicates (n=8). During the experiment, cells were kept warm in their dilutions at 37°C. Replicates were tested one at a time. (b) Results of ROI analysis. Bars depict mean values with standard error of the mean for each concentration and exposure. To increase the visibility of results, a small pop-out window for 95 and 150 ms exposures was created. Values are represented as total counts for measured gray values.

We evaluated the minimal detection settings for our custom setup by titrating cell concentrations in addition to testing different exposure lengths with decreasing numbers of expressing cells [Fig. 3(a)]. In the interest of keeping the frame rates to neurobiologically relevant time scales, the maximum imaging length was set at 2000 ms. Using the imaging processing method described above, we found that we were able to detect a signal above background (mean $\text{total counts}=1.501e^{4}$, $\text{standard error}=3.683e^{3}$) as low as 95 ms for as few as 12,500 cells though 2 mm phantoms [Fig. 3(b)]. At the higher end, we were able to successfully measure populations of 200,000 cells at 95 ms exposures (mean $\text{total counts}=1.313e^{5}$, $\text{standard error}=2.496e^{4}$).

### 3.4 Evaluation of EMCCD Imaging Setup for Depth Comparison

**Fig. 4:**
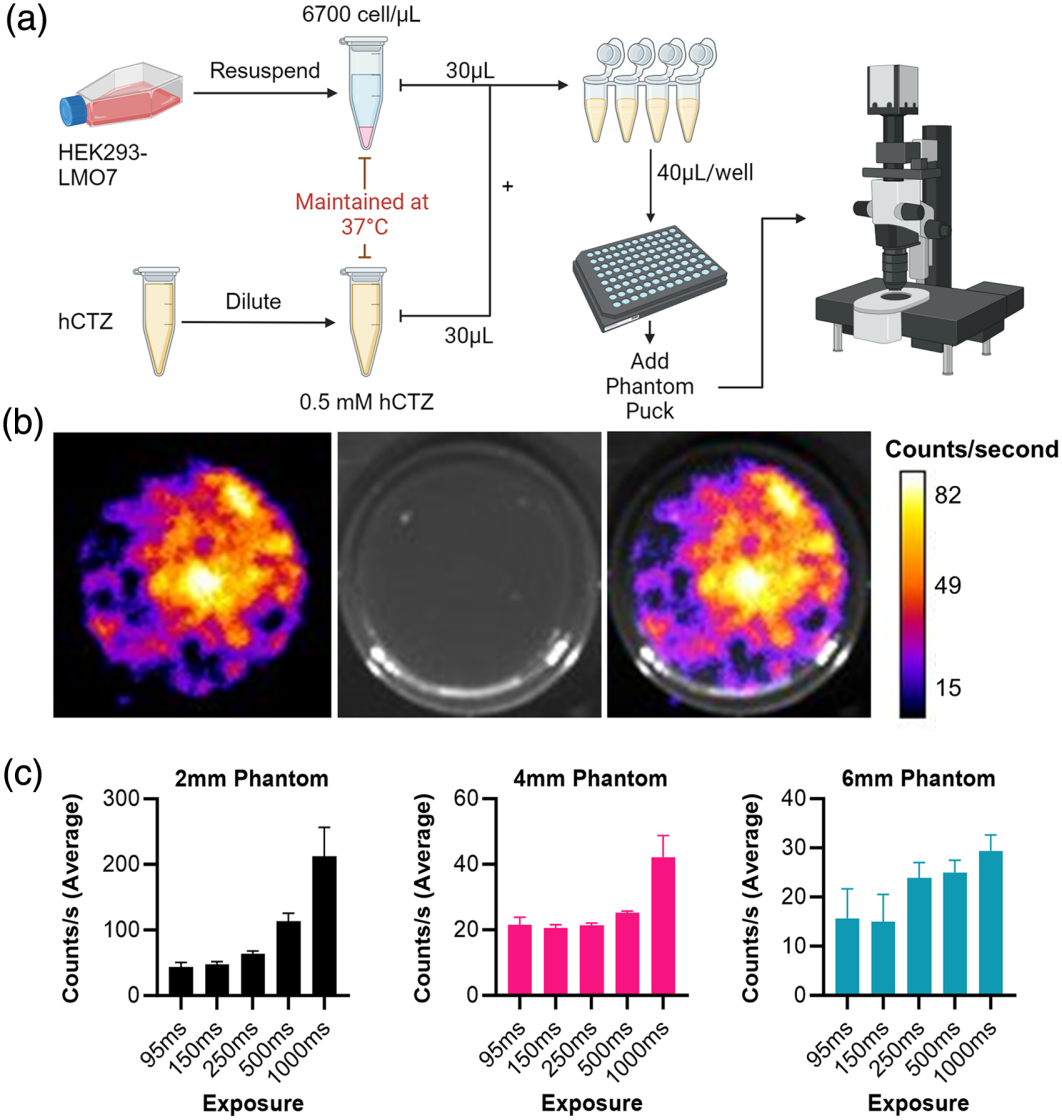
EMCCD recording of bioluminescence through tissue phantom. (a) A diagram of the workflow illustrating the method of bringing the cells from culture to testing. (b) A representative image showing the bioluminescence intensity (right) and brightfield (left) of a 4 mm phantom puck in the 96-well black plate. (c) Measured total photon emission was detected with an Andor EMCCD iXon camera at each exposure time. Each condition was tested in replicates of 3. Counts calculated with averaged blank background subtracted from each recording.

We explored a wide array of imaging technologies. Options included custom microscopes, commercial options, and even lens-less imaging. A secondary goal of this project was the reduction of cost for imaging bioluminescence. We chose to test readily available and ubiquitously used imaging equipment to create our imaging setup: equipping a standard dissection microscope with an EMCCD camera and tube lens adaptor and building a light tight imaging chamber. Recording with this setup followed a format similar to the IVIS experiments. Figure 4(a) shows the workflow for this experiment. Figure 4(b) shows a representative image for a 4 mm phantom at 500 ms exposure. We found that using our setup, it was possible to detect the signal above background beginning at 95 ms exposure times [Fig. 4(c)]. With our equipment, this translated to a recording speed of 10 Hz. The level of brightness above background was significantly greater for all exposures for the 2 mm phantom depth. As expected, 4 and 6 mm phantoms were much less permissive of light, but we were still able to image at 10 Hz. Here we see that it is possible to detect a relevant signal above background.

### 3.5 *In Vivo* Imaging

**Fig. 5:**
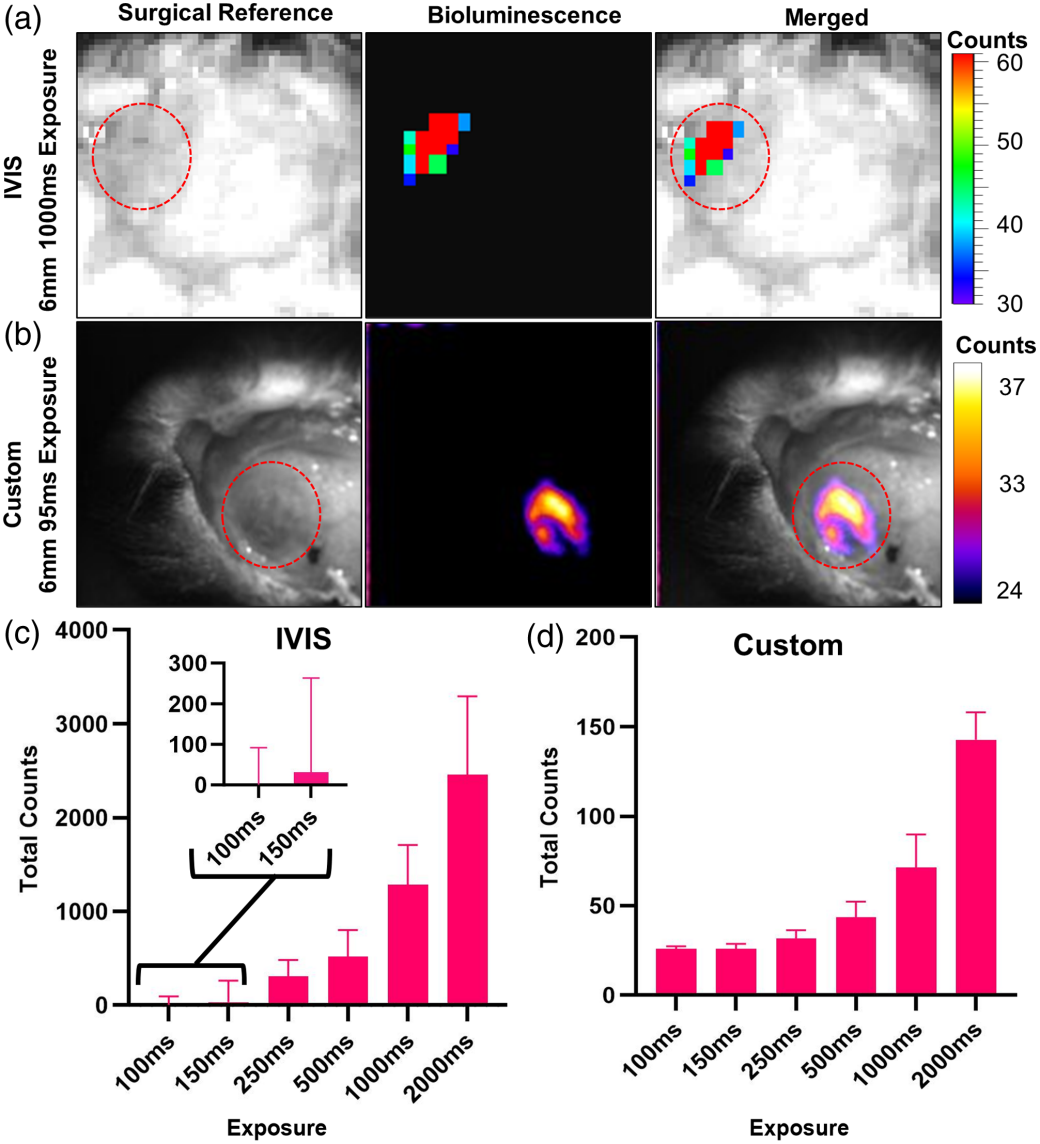
Mice transduced with SSLuc at 4 mm depth imaged with IVIS and EMCCD. Bioluminescence collected on the IVIS (A) and the custom imaging setup (B) shown above. Calibration bars indicate counts per second. (a) Bioluminescence collected on the IVIS imaging system at 1000 ms exposure. (b) Bioluminescence collected through thinned skull with the EMCCD camera at 95 ms exposure time. (c) Total counts of pixels recorded on the IVIS. (d) The same measure collected with the EMCCD camera. Graph shows total counts averaged across animals (n=3). Error bars indicate SEM.

To validate the findings from our *in vitro* model, we transduced mice at 4 mm with AAV9-hSyn-SSLuc [Figs. 5(a) and 5(b)]. Total counts from the selected ROI with the IVIS were generally higher compared with the EMCCD setup when testing longer exposure times [Figs. 5(c) and 5(d)]. However, the IVIS was not able to record a significant signal above background at exposures shorter than 250 ms for either the phantom or animals [Figs. 5(e)]. These results closely match the phantom recordings from the SSLuc stable cell line.

### 3.6 Bioluminescence Microscope Imaging: Bioluminescent Sensor Assay

Seeing that we were able to use the phantom to simulate *in vivo* conditions when using a green light emitting intact luciferase, we next sought to test the phantom with a different luciferase that emits a different wavelength of light. Simultaneously we also sought to demonstrate the utility of the phantom for performing pre-*in vivo* biosensor assays. We chose the bioluminescent indicator of the neurotransmitter glutamate (BLING 1.0), which is based on NanoLuc, emits blue light, and increases in luminescence in the presence of high glutamate levels. It has been applied to report induced seizures *in vivo* at depths beyond what is accessible with fluorescence imaging^35^

For this assay with the blue light emitting luciferase, we used the ideal ink concentration of approximately 0.265% for a phantom matching the absorbance of fresh brain tissue for an emitted wavelength of 470 nm [Fig. 1(b)].

**Fig. 6:**
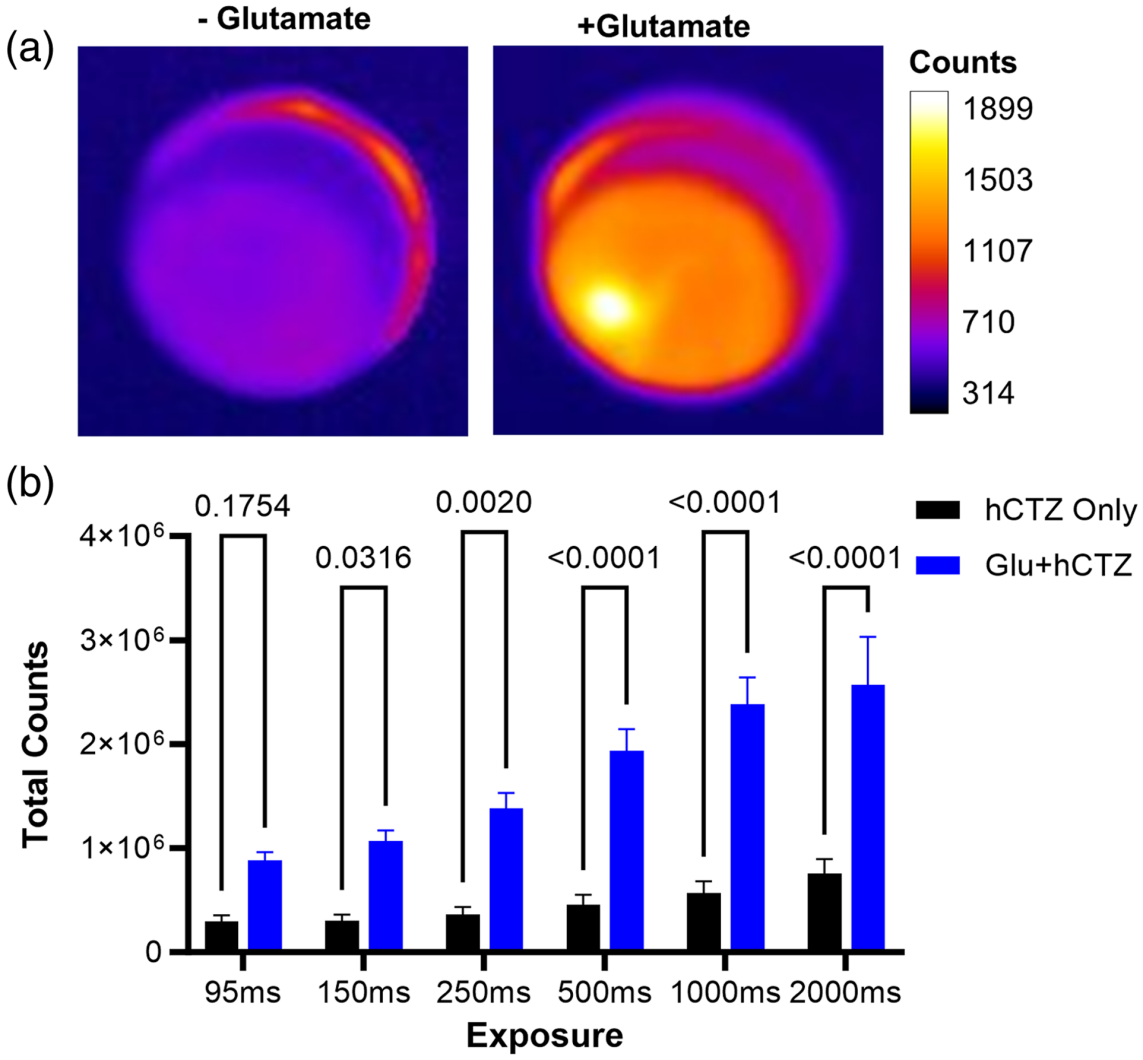
Bioluminescent neurotransmitter reporting through tissue phantom. (a) Side by side depiction of BLING 1.0 expressing cells covered by a 2 mm thick brain phantom puck with or without glutamate. Images are set to scale depicted by calibration bar on left. Values are represented by total counts and color coded with a heat map (look up table: fire). Panels depict an increase in bioluminescent light in response to the presence or absence of glutamate. (b) Quantification of ROI’s. Using a custom script (See Supplementary Material.) ROI’s for each replicate (n=5) in FIJI. Calculated ROI’s for each exposure and with or without glutamate conditions shown in bar graph. 2-Way ANOVA (F=134.8, p<0.001) was used to determine statistical significance of group mean differences.

Visual analysis of glutamate reporting through the phantom rendered clear visual differences between testing conditions without glutamate and with high levels of glutamate [Fig. 6(a)]. The distinction between testing conditions, without and with glutamate became more statistically significant as the exposure time was increased [Fig. 6(b)]. Group means assessed by two-way ANOVA and Bonferroni post hoc revealed that an increased exposure length for similar conditions enhanced the ability to discriminate between background bioluminescence and neurotransmitter reporting via the sensor. The detected counts reporting both baseline activity and glutamate reporting represent bioluminescence measured above noise and dark current. These results highlight the importance of optimizing imaging parameters to reduce optical noise and increase the detection of the true signal at a given exposure time and cell count for a bioluminescent sensor prior to initiating *in vivo* experiments. Further, the adoption of a pre-*in vivo* technique such as this can save researchers time and resources by helping to ensure successful translation to *in vivo* imaging by pre-optimizing imaging parameters.

## 4 Analysis

### 4.1 Absorption Spectra of Phantom Versus Brain Tissue

Absorbance spectra collected using the Tecan Spark was evaluated using GraphPad Prism software. Replicates were checked for percent covariation to evaluate the quality of the collected data. 2-way ANOVA was used to compare optical density (OD) values at 470, 530, 630, and 670 nm.

### 4.2 Estimation of Analogous Ink Concentration

Absorbance data for 470, 530, and 670 nm wavelengths was generated by scanning replicates of varying concentrations but similar depths (2 mm) on a Tecan Spark. Area scans were performed using a 9-point multipoint scan at frequencies for each given frequency. Area scans were averaged for within well values and normalized to wells containing 1× PBS only. Replicates were analyzed for %CV for quality control. All wells had minimal variability and were included in analysis.

### 4.3 IVIS Image Analysis

Initial experiments were done with the IVIS due to its widespread use for imaging bioluminescence. Bioluminescence was measured from ROIs matching the size of the well and expressed as total counts from the included Living Image software. Replicates (n=2) were averaged for a given phantom depth for each exposure time. Values were analyzed with two-way ANOVA and compared with blank wells [Fig. 2(b)].

### 4.4 EMCCD Image Analysis

Background was recorded for each exposure time prior to recording bioluminescence for 100 frames (binning 4×4, Photon Counting preset). Image processing was performed using Fiji. Recorded frames were scanned for visible aberrations, and each frame with a significant artifact, such as a bright line across the frame, was deleted from the stack. A Gaussian blur was applied to each frame of the cleaned stack, and then the stack was averaged into a single image. Averaged dark frames were subtracted from experimental recording Z-stacks for a given exposure time. For example, the background averaged dark image was subtracted from each frame of a 95 ms exposure recording. Files were then filtered for outliers and then z-projected according to the stack average. The well in each image containing a sample was measured with an ROI corresponding to the size of the well. For *in vivo* experiments, ROIs were placed over the entire area of the thinned skull. Replicate values for each depth were averaged across their exposure times. Representative images were pseudo-colored with a lookup table and adjusted in brightness. Calibration bars were generated with Fiji and attached to representative images.

## 5 Discussion and Conclusion

In summary, we have developed a method for optimizing imaging conditions for recording bioluminescent light at a fast frame rate and at significant depth for labs to optimize hardware and imaging parameters prior to initiating *in vivo* experiments. We began by validating the brain tissue phantom for light absorbance compared with fixed and fresh brain tissue, testing different concentrations of absorbance material to validate the phantom’s use and build upon previous work. Each subsequent step of our experiments was tested in both our own microscopy setup and the commercially available IVIS. We next tested a green light emitting luciferase expressed in HEK 293 cells with this brain tissue phantom. These results were validated in mice transduced with the same construct. Bioluminescence imaged through thinned skulls has a brightness similar to the *in vitro* stable SSLuc cells and phantom combination. We were able to record significant bioluminescent activity above background with a high SNR.

In our study, we developed an *in vitro* method of modeling bioluminescent detection. This was accomplished using an empirically validated optical tissue phantom. Part of the incentive to use this phantom was to reduce the number of animals required to determine the ideal imaging conditions for a given bioluminescent construct. A key limitation in the adoption of new bioluminescent reporters is the transition from *in vitro* to *in vivo*. The simple fact is that modeling or creating expectations for the precise effects that tissue will have on the reduction of collected photons for a newly designed construct is not realistic. Thus, functional testing and optimization throughput time can be increased with preliminary testing *in vitro* with simulated *in vivo*-like conditions.

Previous studies have performed activity imaging with bioluminescent light emission recorded through the closed skull by red shifted luciferase/substrate variants. For example, work by Tian et al. generated a bioluminescent calcium sensor capable of reporting neuronal activity through a closed skull. Calcium flux was detected, and seizure activity was reported in real time for areas of the hippocampus and responses to foot shock in the basolateral amygdala (5 mm depth) at a frame rate of 1 Hz (1 s exposure). Oh et al. was able to report calcium fluctuations non-invasively in the liver using their bioluminescent orange calcium sensor. Images for these experiments were captured using the IVIS at 0.5 Hz (2 s exposure). These studies demonstrate that it is possible to detect relevant signals from bioluminescent reporters beneath dense tissue.

When running our experiments using a green-emitting bioluminescent construct (535 nm), we were able to effectively collect sufficient light above background. For imaging fast activity that occurs on time scales shorter than 250 ms, we found that the EMCCD imaging setup showed better sensitivity, recording at 10 Hz, 95 ms exposure plus an ∼5  ms hardware/data transfer delay, whereas in this case, the IVIS successfully recorded at 4 Hz. One of our goals for this study was to effectively record at a frame rate close to or higher than 10 Hz. The discovery of this hardware limitation led us to seek out alternative equipment to incorporate into our new method. Utilizing an EMCCD camera and off-the-shelf microscopy components, we were able to overcome this recording limitation. In doing so, we demonstrated that it is possible to record a lower-than-red wavelength bioluminescent signal through an optically challenging medium at relatively fast frame rates.

To validate that our *in vitro* model could simulate the *in vivo* conditions with reasonable fidelity, we transduced mice at 4 mm with virus containing the SSLuc construct. In this case, we opted for skull thinning because it allows for improved photon escape while still preserving the integrity of the BBB (blood brain barrier); the skull of transduced mice was very carefully thinned prior to imaging. We were able to preserve the integrity of BBB underneath using the condition of the vasculature as a proxy for BBB health. By thinning the skull, we were able to detect signal at exposure times as low as 95 ms. This corresponded to a frame rate of 10 Hz when accounting for the cameras hardware delay of ∼5  ms. We therefore believe that it is reasonable to expect that activity occurring on fast time scales, such as neurotransmitter release, could be detected with bioluminescence in real time when adopting custom built optical setups that we expect to allow for the detection of an order of magnitude more photons per exposure time.^43^^,^^44^ This validation testing further solidified the expectation that this phantom method could be used to enhance a bioluminescent construct development pipeline and adoption of bioluminescence for routine activity imaging in labs while minimizing resources.

A limitation of our method is the simplicity of the phantom itself. As is visible in the absorbance curve of Fig. 1, brain tissue is highly heterogenous with multiple tissue types and the presence of hemoglobin contributing to its light absorbing properties varying with wavelength. The brain tissue phantom is consistent throughout and does not reflect this variability in tissue type. Future directions could explore different ways of layering the agar-based phantom that we used as a part of our study. Additionally, the microscopy setup that we used could likely be improved upon with custom setups that are more permissive to photons. There were multiple lenses between the EMCCD sensor and the bioluminescent light source itself, including the use of a zoom lens. This likely reduced the overall signal due to the loss of photons as they traveled through the imaging path. Other groups have employed custom built microscopes that we expect would be capable of achieving high frame rate bioluminescent light recordings.^43^^–^^45^ A caveat to these designs is that they require calibration and a level of expertise in construction that many may not have the time or resources necessary to effectively utilize for the initial adoption of bioluminescence imaging. For this reason, we chose to use off-the-shelf components including a pre-assembled dissection microscope for this study and expect to improve on our results going forward.

## Supplementary Material



## Data Availability

All data are available upon request.
